# Arm Crank and Wheelchair Ergometry Produce Similar Peak Oxygen Uptake but Different Work Economy Values in Individuals with Spinal Cord Injury

**DOI:** 10.1155/2016/5481843

**Published:** 2016-04-10

**Authors:** Tom Tørhaug, Berit Brurok, Jan Hoff, Jan Helgerud, Gunnar Leivseth

**Affiliations:** ^1^St. Olav's University Hospital, Department of Physical Medicine and Rehabilitation, Spinal Cord Injury Unit, 7006 Trondheim, Norway; ^2^Faculty of Medicine, Department of Neuroscience, Norwegian University of Science and Technology, 7491 Trondheim, Norway; ^3^Faculty of Medicine, Department of Circulation and Imaging, Norwegian University of Science and Technology, 7491 Trondheim, Norway; ^4^Hokksund Medical Rehabilitation Centre, 3300 Hokksund, Norway; ^5^Department of Sports and Outdoor Life Studies, Telemark University College, 3800 Bø, Norway; ^6^Institute of Clinical Medicine, Neuromuscular Disorders Research Group, The Arctic University of Norway UiT, 9019 Tromsø, Norway

## Abstract

*Objective.* To study whether values for peak oxygen uptake (VO_2peak_) and work economy (WE) at a standardized workload are different when tested by arm crank ergometry (ACE) and wheelchair ergometry (WCE).* Methods.* Twelve paraplegic men with spinal cord injury (SCI) in stable neurological condition participated in this cross-sectional repeated-measures study. We determined VO_2peak_ and peak power output (PO_peak_) values during ACE and WCE in a work-matched protocol. Work economy was tested at a standardized workload of 30 Watts (W) for both ACE and WCE.* Results.* There were no significant differences in VO_2peak_ (mL·kg^−1^·min^−1^) between ACE (27.3 ± 3.2) and WCE (27.4 ± 3.8) trials, and a Bland-Altman plot shows that findings are within 95% level of agreement. WE or oxygen consumption at 30 W (VO_2-30W_) was significantly lower during WCE compared to ACE (*P* < 0.039). Mean (95% CI) PO_peak_ (W) were 130 (111–138) and 100 (83–110) during ACE and WCE, respectively.* Conclusion.* The findings in the present study support the use of both ACE and WCE for testing peak oxygen uptake. However, WE differed between the two test modalities, meaning that less total energy is used to perform external work of 30 W during wheelchair exercise when using this WCE (VP100 Handisport ergometer). Clinical Trials Protocol Record is NCT00987155/4.2007.2271.

## 1. Introduction 

Individuals with spinal cord injury (SCI) live an involuntary sedentary lifestyle that may result in reduced muscle mass and fat accumulation [1–3], impeding performance of exercises to improve cardiovascular health. Indeed, one of many negative health effects of SCI is an increased prevalence of cardiovascular disease (CVD), which is the leading cause of early death in this patient group [4, 5]. To prevent the possible development of CVD, it is mandatory that SCI individuals exercise with adequate training intensity in the same manner as able-bodied individuals to enhance and maintain cardiovascular fitness [6]. It is therefore important that training intensity is adjusted to individual exercise capacity as defined by peak oxygen uptake (VO_2peak_) and power output (PO).

VO_2peak_ is a valid and sensitive outcome measure for assessing physical capacity in both able-bodied [7] and SCI individuals [5]. It should be a reliable determinant of cardiovascular fitness and a major predictor in the evaluation and treatment of the heightened CVD morbidity and overall mortality [5] for able-bodied [7] and SCI individuals. VO_2peak_ and power output (PO) in SCI individuals are most often tested using either arm crank ergometry (ACE) or wheelchair ergometry (WCE). It has been suggested that ACE is less strenuous than WCE [8–11]; however, the determination criteria for VO_2peak_ measurements using ACE or WCE are inconsistent, resulting in disparate values. Sawka et al. [9] reported lower VO_2peak_ and heart rate (HR) using ACE, while Hintzy et al. [12] found higher VO_2peak_ and PO_peak_ values using ACE. Alternatively, Arabi et al. [13], Martel et al. [8], and Glaser et al. [14] all found no difference in VO_2peak_ between ACE and WCE, while Hettinga and Andrews [15] reported that WCE resulted in higher VO_2peak_ values. In addition to different determination criteria, these disparate VO_2peak_ and PO values reported using ACE and WCE may reflect population heterogeneity and methodological diversity among studies.

Guidelines for improving cardiovascular aerobic fitness in SCI have been published [2, 16, 17]. Although recommendations on how to perform VO_2peak_ tests in SCI individuals exist [8, 18], standardized determination criteria for a true VO_2peak_ are still lacking. As previous studies have examined SCI individuals under different testing conditions (equipment and training intensities), it is of clinical value to test whether VO_2peak_ and peak power output (PO) values derived from ACE and WCE are interchangeable. Therefore, ACE and WCE require more experimental research to reveal task-specific differences in cardiovascular capacity as to provide exercise recommendations. Although power output (PO) dependent gross mechanical work efficiency (ME; ratio of PO to energy expenditure) is a valid measure of overall improvement [19], peak power output and mechanical efficiency issues are complex and prone to overestimation [20]. Indeed, physical capacity varies considerably among SCI individuals, suggesting that standardized VO_2peak_ and power output measurements are further developed to obtain comparative values for clinical practice and research [12, 21]. The collection of biomechanical and physiological data during wheelchair propulsion and arm ergometry should be performed on validated and calibrated medical equipment.

The main aim of this study was to investigate whether task-specific differences exist between ACE and WCE when VO_2peak_ is measured using standardized and comparable test determination criteria. In addition, measurements were performed at 30 Watt (VO_2-30W_) to examine whether there are differences in oxygen consumption required to perform external work (WE) between ACE and WCE.

## 2. Materials and Methods

### 2.1. Participants

Twelve male SCI individuals with sensory-motor complete injury (American Spinal Cord Injury Association Impairment Scale A (AIS A) to sensory-motor incomplete AIS C) were enrolled, all in a stable neurological state (Table 1). None of the participants were wheelchair athletes or were using performance enhancing or reducing (e.g., beta-blockers) drugs. None of the SCI individuals used abdominal binders or antithrombolytic stockings during testing. Candidate participants with a pacemaker, cancer, decubital ulcers, a medical history of unexpected autonomic dysreflexia, gross joint contractures, or acute shoulder girdle or joint tendonitis were excluded.

The study was approved by the Regional Committee for Medical Research Ethics and all participants provided informed consent prior to participation. We certify that all applicable institutional and governmental regulations concerning the ethical use of human volunteers were followed and that all procedures conformed to the latest revision of the Declaration of Helsinki.

### 2.2. Test Equipment and Measurements

**Figure 1:**
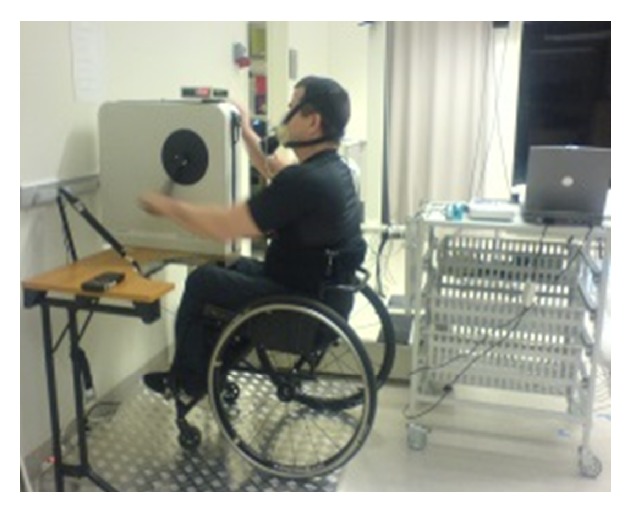
The ACE experimental setup.

**Figure 2:**
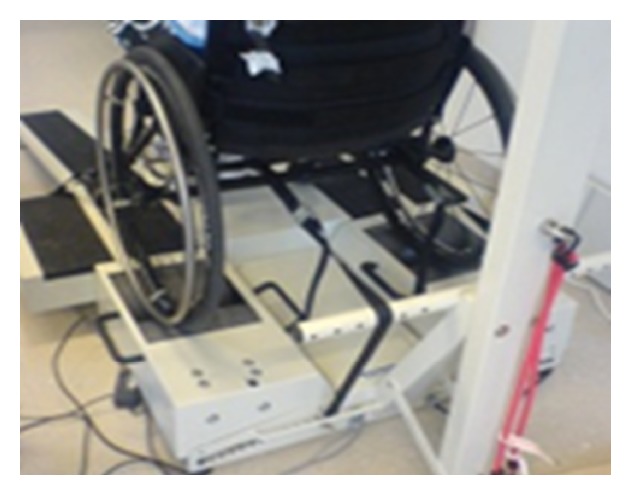
The WCE experimental setup.

An electromagnetically braked Ergomed 840 L (Siemens, Germany) was modified for asynchronous ACE. For WCE, the VP100 Handisport (Medical Development, France) was used as it has been shown to yield reproducible VO_2_ measurements [22]. For WCE, the VP100 Handisport (Medical Development, France) (Figure 2) was used as it has been shown to yield reproducible VO_2_ measurements [22]. For both ACE and WCE testing, all participants used their own rigid-frame wheelchair (Figures 1 and 2).

Prior to all tests, the ACE was calibrated according to the manufacturer's instructions (Siemens Ergomed Operation Manual, 1985) by bringing the ergometer to 90 revolutions per minute (rpm) before applying a braking force and measuring the time required for the rpm to decline to a given lower target. Specifically, the breaking time was correct to 35 rpm in 40 s with a 0 W braking load and to 0 rpm in 18 s using a 25 W braking load. To achieve horizontally aligned shoulder joint axes during ACE, wheelchairs were positioned on a steady platform and the elbows positioned slightly flexed at the point of furthest reach. The WCE equipment was calibrated by recording the total frictional rolling resistance (residual torque/moment of inertia) of zero load with participants in the normal sitting position and the wheelchair attached to the WCE [22].

All ventilation parameters and pulmonary gas exchange measurements were performed using the Metamax II Cortex ergospirometry system (Cortex Biophysik GmbH, Germany). A head cap assembly with facemask, volume transducer, and assembly tube for O_2_ and CO_2_ sensors was fixed on the participants during all tests. The volume range and accuracy were 0.01−14.0 L·s^−1^ and 1.5%, respectively. The oxygen concentration range and accuracy were 0−25 vol.% and <0.1 vol.%, respectively. CO_2_ levels were analysed by an infrared sensor with a range from 0 to 10 vol.% and accuracy of <0.1 vol.%. The volume transducer was calibrated with a 3-L standardized calibration syringe (Hans Rudolph Jäger GmbH, Germany). The gas concentration sensors were calibrated with ambient air and a chemically standardized calibration gas comprised of 16% O_2_, 4% CO_2_, and 80% N_2_ (SensorMedics Corporation, USA).

Blood lactate concentration ([La^−^]_b_ (mM)) was measured within one minute after termination of VO_2peak_ tests with an accuracy of ±3% using the Lactate Pro Analyzer LT-1710 (Arkray Factory, Inc., KDK Corp., Japan). Heart rate (HR) was measured with Polar® watches (Polar Electro, Oy, Finland) during all tests with accuracy of ±1 heart beat (Polar Operation manual, Polar Electro, 1997). For the subjective evaluation of perceived fatigue during tests, a rating scale of perceived exertion (RPE) from 6 to 20 (Borg 1970) was recorded during the last minute of the test.

Work economy (WE) was defined as oxygen consumption (mL·kg^−1^·min^−1^) divided by external work output (W) during steady state submaximal work. A constant 30-W workload (VO_2-30W_) was used during WE measurements for both ACE and WCE to ensure submaximal aerobic conditions.

Mechanical efficiency (ME) was defined as external work output (W) divided by oxygen cost (mL·kg^−1^·min^−1^). VO_2_ and work output were converted to kcal (Kcal · min^−1^) to allow work economy to be expressed as percentage change.

### 2.3. Test Protocol

To minimize carry-over and order effects, the participants were randomly assigned to ACE or WCE as the first testing condition and there was a minimum 24-hours between tests. The test protocol for both ACE and WCE included: (1) a 6-min warm-up period; (2) VO_2-30W_ testing for 4 min; (3) an individualized ramp protocol where work load was increased in steps varying from 5 W to 15 W (depending on the individual) in 1-min intervals, to reach VO_2peak_ as suggested by Froelicher [23]; (4) blood lactate ([La^−^]_b_) was measured within 1 min after termination of the peak test. The following determination criteria were used for VO_2peak_ [23]; Combined with a respiratory exchange ratio (RER) of ≥1.1, [La^−^]_b_ ≥ 7, and RPE ≥ 15 (Borg 6−20), VO_2peak_ was considered achieved. In the upper-body mode a VO_2_ plateau (a VO_2_ plateau, despite an increase in power output, and pulmonary ventilation) is rarely reached, therefore VO_2peak_ is used to denote maximal effort. If these criteria were met, the average of the highest VO_2_ values within three consecutive 10 s measurements was calculated as VO_2peak_.

### 2.4. Statistical Analysis

The nonparametric Wilcoxon signed-rank test was used to compare parameters between test conditions. Similarity was tested using Bland-Altman plot. Values are expressed as mean (standard deviation) or median (range). Significance was accepted at *P* < 0.05.

## 3. Results

**Figure 3:**
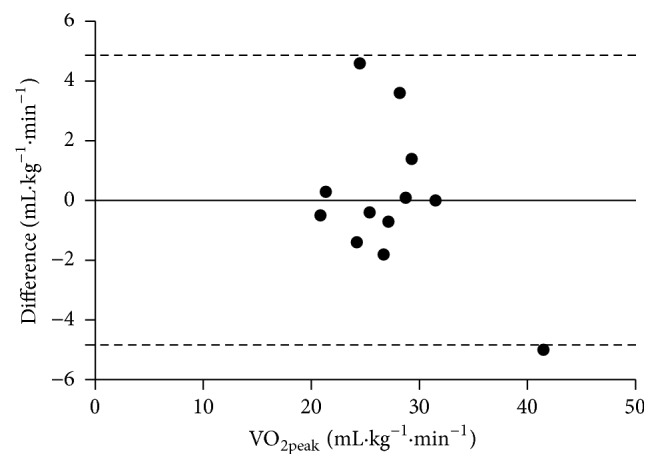
Bland-Altman plot shows differences in VO_2peak_ (mL·kg^−1^·min^−1^) between ACE and WCE within 95% of level of agreement.

**Figure 4:**
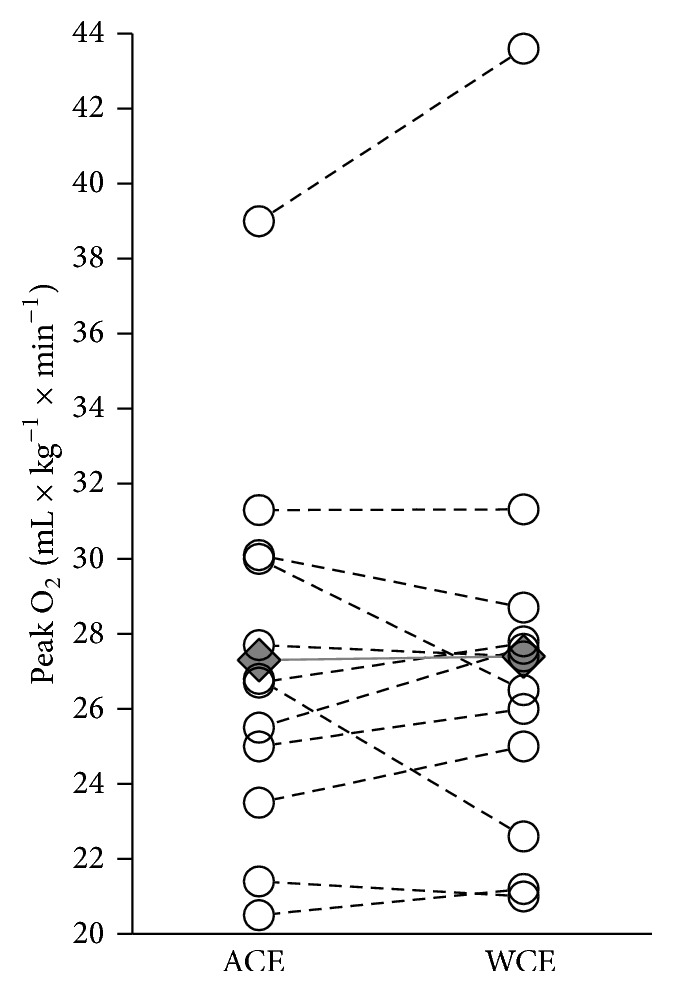
VO_2peak_ (mL·kg^−1^·min^−1^) performance data for all participants in the ACE and WCE modalities. Open circles correspond to individual values; diamonds correspond to median values. *N* = 12.

**Figure 5:**
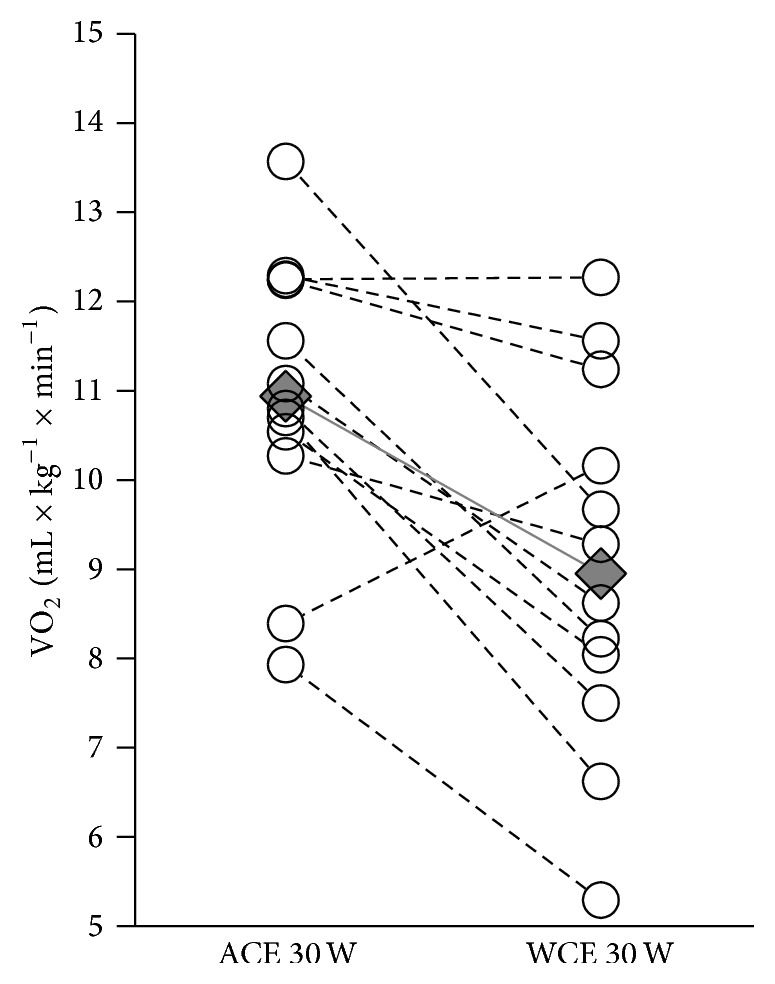
Steady state VO_2_ (mL·kg^−1^·min^−1^) submaximal (30 W) data for all participants in the ACE and WCE modalities. Open circles correspond to individual values; diamonds correspond to median values. *N* = 12.

The participants completed all tests without reporting severe fatigue, shoulder joint, or shoulder girdle pain. No observations on clinically relevant submaximal or peak heart rate disturbances were observed during the course of this study. The Bland-Altman plot shows 11 out of 12 points within the 95% level of agreement and thus a method similarity (Figure 3). There were no significant differences in VO_2peak_, peak HR, peak VE, and peak RER values between ACE and WCE trials (Table 2 and Figure 4). At 30 W, VO_2_ was 22% higher in ACE compared to WCE (*P* < 0.039; Table 2 and Figure 5), indicative of lower WE, and was associated with significantly higher HR and RPE. ACE generated a significantly higher (*P* < 0.001) peak power output (PO_peak_) compared to WCE. In addition, [La^−^]_b_ was significantly higher during ACE. In the VO_2peak_ tests, two participants did not reach the determination criterion of [La^−^]_b_ ≥ 7 mM. However, excluding these two participants from the analysis did not influence the results. For the remaining 10 SCI individuals, median [La^−^]_b_ was still significantly higher following ACE compared to WCE (10.5 (7.9−14.9) mM versus 9.0 (7.4−10.6) mM; *P* ≤ 0.05). Thus, in our statistical analysis, all 12 SCI individuals were included (Table 2).

## 4. Discussion

The main finding in the present study is that there is no significant difference between the two testing modes in terms of testing VO_2peak_. This does not imply that the two methods are analogous, but rather that they may be equally appropriate for determining VO_2peak_ in individuals with SCI [14, 24]. Even if ACE and WCE show similar values for VO_2peak_, the main muscles used to exert work seem different between the two modalities, that is, task specificity. Traditionally, VO_2max_ determination of able-bodied individuals is limited by cardiac output whereas for small muscle groups and not weight bearing activities as in ACE and WCE the limitations are primarily linked to the muscles' aerobic capacity. This makes the use of ACE or WCE and eventual differences between the working modes to a higher degree dependent upon the trained state of the muscles involved in the two different working modes. WE were lower using ACE, PO_peak_, and HR higher, and ACE trials elicited greater subjective exertion ratings. Thus, ACE and WCE seem not to be comparable for submaximal levels of energy cost.

Mean VO_2peak_ values measured in this study were higher than in previous studies (ACE: 27 mL·kg^−1^·min^−1^ versus 19 mL·kg^−1^·min^−1^, [13]; WCE, 27 mL·kg^−1^·min^−1^ versus 21 mL·kg^−1^·min^−1^, [25]), possibly due to population heterogeneity (i.e., the inherent VO_2peak_ levels of the study population). However, such differences may also reflect the lack of standardized determination criteria for VO_2peak_ in previous studies. Indeed, few studies have reported normative WCE-specific SCI VO_2peak_ values. According to Janssen et al. [26], WCE performance in our study cohort indicates average to good cardiovascular fitness. However, the aforementioned study [26] may have been somewhat biased since 40% of the SCI individuals were wheelchair athletes. On the basis of our findings, it appears that the SCI individual fitness levels from earlier studies may have been underestimated, underscoring the importance of defining a true VO_2peak_ via standardized determination criteria.

In the present study, the WE (VO_2-30W_) was significantly higher during ACE compared to WCE (Table 2, Figure 5). The WE was approximately 20% lower on the WCE than using the ACE. A low oxygen cost is indicative of higher % work efficiency. This result contradicts earlier studies comparing VO_2_ measurements by ACE and WCE, which reported ACE to be more efficient and effortless than WCE in terms of mechanical work efficiency (ME; work output divided by energy expended) [9, 27, 28]. The approximate ME for SCI individuals was reported to be 6% during WCE [11, 29] and 15% during ACE [30]. In the present study the ME is 14% and 21% for ACE and WCE, respectively. This may reflect differences in upper-body muscle activity elicited by ACE and WCE. Asynchronous ACE involves a 360° continuous push-and-pull application of force, while the pushing motion in WCE is discontinuous because of hand relocation between pushes. Thus, ACE might be considered a less complex and more continuous upper-body activity than WCE. Other differences may be pertinent as well. Most important is that the ergometers are completely different. The aforementioned studies have been using a different ergometer from the VP100 Handisport used in this comparison. It is difficult to compare the oxygen cost of work output across ergometers. In addition, the speeds of movement/cadence during the 30 W epochs differed between modalities; ACE was fixed at 70 rpm, while the mean optimal speed chosen by SCI individuals during WCE was 52 rpm. This may have resulted in velocity-dependent loss of efficiency during ACE. Indeed, the SCI individuals reported greater subjective fatigue and exhibited greater peak HR on ACE trials compared to WCE trials. The SCI individuals in the present study are also habituated to hand rim-propelled wheelchairs as an act of long-term daily mobility but are relatively inexperienced with asynchronous ACE, which may have further increased the difference in efficiency between ACE and WCE trials. Overall the differences in both ME and WE are considered to be limited to the particular ergometers used.

PO_peak_ values were significantly higher during ACE compared to WCE. Hintzy et al. [12] reported both higher VO_2peak_ and PO_peak_ values for ACE relative to WCE in able-bodied individuals, and Glaser et al. documented similar findings when comparing SCI to able-bodied individuals [14]. It has been suggested that physiological adaptations of the intact upper-body musculature in experienced paraplegic wheelchair ambulatory individuals contribute to this effect [14, 31]. Walker et al. [18] argued that an individualized high-intensity testing protocol (20 W/min increments per interval) elicits higher PO_peak_ and VO_2peak_ values. The mean PO_peak_ values during ACE and WCE reported in a recent SCI review, 85 W and 75 W, respectively [21], are lower than the values measured in the current study (130 W and 100 W), suggesting that our SCI individuals elicit a higher aerobic work performance. Another explanation may be that less of the total upper-body power production during WCE is transmitted to the ergometer, and therefore the ME seems reduced compared to ACE. Finally, Stewart et al. [24] obtained more reliable values for VO_2_, HR, and RPE, with higher intraclass correlation coefficients in peak testing compared to submaximal testing. A lower reliability during submaximal tests and a reduced ME in WCE, therefore, may cause some of the measured differences in this study.

Alternatively, our findings are also comparable in several respects to studies [8, 14, 28] reporting lower ME and PO_peak_ values in WCE. Lower PO_peak_ values in WCE may be due to the fact that less of the total upper-body power production is transmitted to the ergometer, and therefore the MWE appears lower compared to ACE. A peak power loss of up to 30% has been calculated for WCE compared to ACE [8, 12, 14, 32]. Interestingly, adding 30% to our PO_peak_ WCE values brings the total PO to approximately 130 W, equalling that of ACE. In addition, the identical mean VO_2peak_ values for both ACE and WCE demonstrate that the total energy demands are identical despite the 30% lower PO_peak_ values during WCE [8, 24].

When blood lactate production ([La^−^]_b_) exceeds [La^−^]_b_ elimination, the anaerobic threshold is reached and muscle fatigue may occur [24, 31, 33]. In our study, peak values for HR and [La^−^]_b_ were significantly lower during WCE, indicating that [La^−^]_b_ accumulation in the blood occurred less rapidly than during ACE. This may be due to an inherent task-specific difference in upper-body capacity between WCE and ACE. These task-specific muscular characteristics were demonstrated by Schneider et al., who reported that the ACE [La^−^]_b_ threshold occurred at 58.9% of VO_2peak_ in SCI individuals compared to 50% of VO_2peak_ in able-bodied individuals [31]. The differences observed in our study indicate that the lactate [La^−^]_b_ threshold in WCE may occur at a higher percentage of VO_2peak_ than in ACE. Thus, the specific upper-body muscles involved in ACE and WCE appear different even if the total recruitable upper-body muscle mass seems to be similar. Subsequently, there is most probably the total muscle mass in use that limits the VO_2peak_. Therefore, the lower [La^−^]_b_ during WCE may reflect underlying differences in both the training specificities and muscular movement characteristics of the two modalities [34, 35], resulting in difficulty recruiting upper-body muscle mass during WCE compared to ACE [36].

The use of accurate VO_2peak_ determination criteria is critical in defining true VO_2peak_ values. Our main ACE and WCE VO_2peak_ determination criteria are a respiratory exchange ratio (RER) of ≥1.1, [La^−^]_b_ ≥ 7, and RPE ≥ 15 (Borg 6−20). By including these criteria, the accuracy of measuring VO_2peak_ may be enhanced. Moreover, these criteria may increase between-study comparability since VO_2peak_ values obtained by ACE are comparable to those obtained by WCE.

In conclusion, for VO_2peak_ testing, the two methods appear equivalent. The trained state of the muscle groups used for the one working mode over the other might however lead to differences. The SCI individuals in this experiment were used to both working modes, indicating that the amount of muscle mass involved seems to be relatively similar. However, if work economy is tested, ACE and WCE cannot be used interchangeably and seem to be highly dependent upon the ergometer used.

## Figures and Tables

**Table 1:** Characteristics of SCI individuals included in the present study.

Subject	LOI/AIS	Age (y)	Ht (m)	Mass (kg)	TSI (y)
1	Th8/A	48	1.68	78	33
2	Th12/B	60	1.86	79	10
3	Th5/A	45	1.88	81	29
4	Th3/A	49	1.86	95	23
5	Th11/A	43	1.85	126	12
6	L1/A	35	1.78	78	11
7	L1/C	45	1.86	67	11
8	Th9/A	39	1.96	97	13
9	Th4/C	31	1.70	66	4
10	Th8/A	55	1.80	72	24
11	Th9/A	52	1.96	77	36
12	Th11/A	62	1.80	70	21

	Median	46.5	1.86	78.0	22
	Range	(31)	(28)	(60)	(26)

LOI: level of injury; AIS: American Spinal Injury Association Impairment Scale grade; Ht: height; TSI: time since injury.

**Table 2:** Submaximal (VO_2-30W_) and peak physiological values during arm crank ergometry (ACE) and wheelchair ergometry (WCE) presented as mean and confidence interval (CI) values.

Variables	ACE 30 W (*n* = 12)	WCE 30 W (*n* = 12)	ACE peak (*n* = 12)	WCE peak (*n* = 12)
VO_2_ (mL·kg^−1^·min^−1^)	10.9 (9.9–11.9)	9.0 (7.7–10.3)^*∗*^	27.3 (24.1–30.5)	27.4 (23.6–31.2)
VO_2_ (L·min^−1^)	0.87 (0.83–0.92)	0.72 (0.64–0.79)^*∗*^	2.20 (1.98–2.42)	2.20 (1.96–2.44)
VE (L·min^−1^)	24.2 (22.8–25.7)	18.7 (17.7–22.8)^*∗*^	95.7 (81.7–101.4)	93.9 (75.3–99.4)
RER	0.92 (0.88–0.94)	0.91 (0.87–0.93)	1.19 (1.16–1.25)	1.17 (1.11–1.23)
[La^−^]_b_ (mmol·L^−1^)			11.3 (9.1–13.4)	8.5 (7.6–9.3)^*∗*^
HR	110 (56)	95 (85–109)^*∗*^	179 (168–185)	173 (156–183)
RPE	10 (8–10)	7 (6–8)^*∗*^	18 (17-18)	17 (16–18)
Workload (W)	30	30	130 (111–138)	100 (83–110)^*∗*^

ACE 30 W: ACE at submaximal effort (VO_2-30W_).

ACE peak: ACE at maximal effort (VO_2peak_).

WCE 30 W: WCE at submaximal effort (VO_2-30W_).

WCE peak: WCE at maximal effort (VO_2peak_).

VO_2_ (mL·kg^−1^·min^−1^): Oxygen uptake.

VO_2_ (L·min^−1^): Oxygen uptake.

VE (L·min^−1^): Pulmonary ventilation.

RER: Respiratory exchange ratio.

[La^−^]_b_ (mmol·L^−1^): Non-hemolyzed blood lactate concentration.

HR: beats.·min^−1^.

RPE: Rating of perceived exertion.

Workload: Power output in Watt.

Workload at peak: PO_peak_ in Watt.

^*∗*^Level of significance *P* < 0.05.

## Abbreviations

VO_2peak_: Peak oxygen uptake
ACE: Arm crank ergometry
WCE: Wheel chair ergometry
WE: Work economy
PO_peak_: Peak power output
PO: Power output
VE: Pulmonary ventilation
RER: Respiratory exchange ratio
RPE: Rating of perceived exertion
SCI: Individuals with spinal cord injury
CVD: Cardio vascular disease
HR: Heart rate
[La^−^]_b_: Blood lactate concentration
VO_2-30W_: Constant 30 W workload
ASIA: American Spinal Cord Injury Association
AIS: ASIA Impairment Scale
ME: Mechanical efficiency.
